# Treatment of acute urticaria with acupuncture

**DOI:** 10.1097/MD.0000000000021093

**Published:** 2020-07-17

**Authors:** Shuai Zhou, Ruirui Zhao, Mingxin Xue

**Affiliations:** a Affiliated Hospital of Nanjing University of Traditional Chinese Medicine; b Department of Graduate School, Nanjing University of Chinese Medicine; c Department of Acupuncture, The First Affiliated Hospital of Nanjing Medical University, Nanjing, Jiangsu, China.

**Keywords:** acupuncture, acute urticaria, case report

## Abstract

**Rationale:**

Urticaria is a refractory dermatosis with long duration and a high recurrence rate. More to the point, medication of acute urticaria always demands high doses, which may cause some adverse effects. Acupuncture, with a history for over 2000 years, has been utilized in clinical practice as an alternative treatment strategy for dermatologic diseases.

**Patient concerns:**

A 26-year-old male nurse on duty suddenly fell sick during the night-shift in the ward. Wheals began spreading all over his body, but he had no urticaria medicine on hand. The unbearable itching made it extremely difficult for him to resume working.

**Diagnosis:**

The patient was diagnosed with acute urticaria according to the symptoms.

**Interventions:**

A 30-minute acupuncture treatment was performed.

**Outcomes:**

About 5 minutes after needles were inserted into the acupoints, the patient felt significantly relieved of itching sensation; moreover, he could even subjectively control himself from scratching and could calmly wait for gradual disappearance of the wheals. Then 30 minutes later, the wheals almost faded away.

**Lessons:**

The results suggest that acupuncture may be a promising alternative therapy to treat acute urticaria, especially for patients who cannot receive drug treatment.

## 1 Introduction

Acute urticaria, a skin disease, is a localized edema reaction resulting from dilation and increasing permeability of skin and mucosal small blood vessels. Nearly 20% of people get acute urticaria at least once in their lifetime. This disease is mainly manifested with wheals in various sizes and unendurable itching.^[1–3]^ At present, clinical treatment methods are relatively limited and mainly drug dependent, with a major therapeutic goal of controlling symptoms and improving patients’ life quality. Eliminating inducements or suspicious causes is conducive to the natural disappearance of urticaria.^[4]^ As for the treatment of acute urticaria, the 2nd-generation nonsedating antihistamine is the 1st choice, and glucocorticoids could be adopted if symptoms cannot be effectively controlled.^[5]^ As a nonpharmaceutical therapy, acupuncture has very few adverse effects and is extensively utilized in urticaria treatment in Asia.^[6–9]^ According to the collected data, no relevant clinical report over urticarial treatment through acupuncture on Yuji point (LU1O) has been reported. In this report, we describe a 26-year-old male nurse on duty who got acute urticaria and treated with acupuncture alone in the absence of medication, achieving good curative effects.

## 2 Case presentation

**Figure 1:**
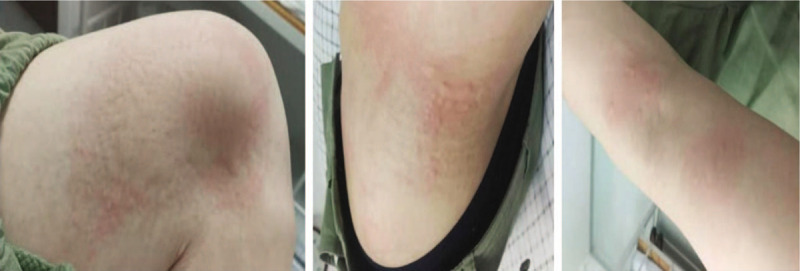
Wheals gradually swelled higher than the skin and spread to the knee joint (A), side waist (B), and especially the elbow part (C).

The patient was a 26-year-old male nurse. During the night shift on June 11, 2019, the nurse suddenly felt itchy on the elbows and waist at 3 am. Scratching could not alleviate itching, and wheals gradually swelled up and spread to the knee joint, side waist and especially the elbow part. The itching was unbearable and agonizing (Fig. 1). The nurse was physically healthy and did not have any underlying disease.

### 2.1 Examination

The medical history of the nurse showed that there was neither any history of relevant skin diseases nor any history of allergies to food or drugs. The onset cause was unclear without any obvious inducement. Given the patient's symptoms and urticaria activity score (UAS) utilized to evaluate wheals and itching, he was diagnosed with acute urticaria.UAS: 4 points.

### 2.2 Intervention

The nurse received acupuncture immediately for once and was informed of the detailed treatment procedures.

Due to quite a few body parts showing symptoms, it was difficult to realize local acupoints selection. As a result, we adopted the “Five Shu points” (including Jing-well point, Xing-spring point, Shu-stream point, Jing-river point, and He-se point) selection method in treatment. According to traditional meridian theory, Xing-spring point Yuji point (LU10) on Lung meridian was chosen.

**Figure 2:**
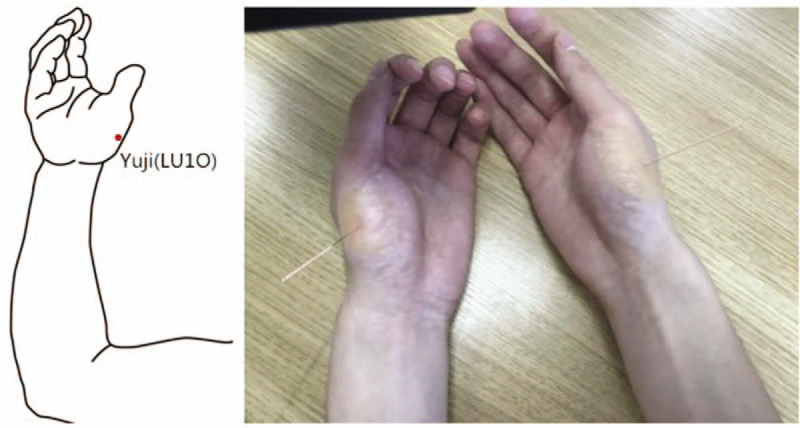
The operator stabbed the needles into the bilateral Yuji point (LU10) on the patient's hands.

Upon disinfection of the operator's hands and the acupoints, 2 disposable stainless needles (acupuncture needle with a diameter of 0.3 mm and length of 40 mm provided by Suzhou Huatuo Medical Products Factory Co Ltd, Suzhou, China) were stabbed into the bilateral Yuji (LU10) on the patient's hands with the depth of 10 mm (Fig. 2). After the needles were inserted, the needles were lifted, thrust or rotated to obtain De qi sensation. The above manipulation was repeated every 5 minutes, and the needle was retained for 30 minutes in total.

### 2.3 Outcome

**Figure 3:**
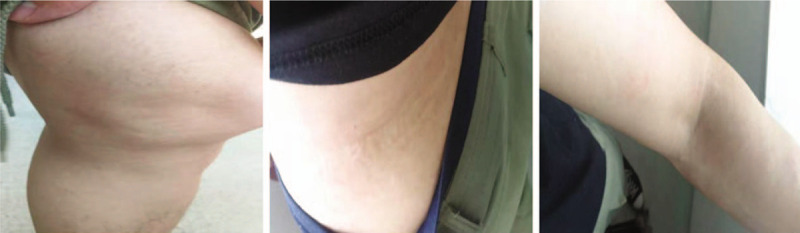
The wheals gradually disappeared at the knee joint (A), the lateral waist (B), and the elbow (C).

After 5 minutes of needling, the itching sensation was significantly relieved, and the patient could subjectively control himself from scratching. The wheals gradually disappeared at the knee joint, the lateral waist, and the elbow. After 30 minutes, the wheals practically vanished (Fig. 3).

## 3 Discussion

Over recent years, acupuncture, as an antipruritic measure, has been broadly applied in the treatment of dermatosis.^[10]^ As proved by relevant literatures, urticaria is a self-limited disease, and acute urticaria could commonly get relieved even without active intervention.^[5]^ In this case, the patient with the initial onset of acute urticaria occurred during his night-shift work accompanied with unbearable itching and restlessness. In the absence of medication, acupuncture treatment retarded the disease progress within a short period of time, displaying an outstanding therapeutic performance and indicating that acupuncture at the LU10 is effective in acute urticaria treatment.

Based on the traditional Chinese medicine theory, the “wind” is the fundamental pathogenesis of urticaria, referring to some external physical factors (friction, pressure, coldness, heat, sunlight, etc). Acute urticaria is commonly featured by wind-induced diseases^[11]^; therefore, its treatment should be centered on expelling wind evil. There is an ancient saying that “to treat wind, 1st treat the blood, when the blood moves, wind naturally disappears.”^[12]^ There is a theory that “lungs govern skin and hair,” that is, the essence of skin and hair is nourished and warmed by lungs; meanwhile, respiration of skin and hair as well as opening and closing of the sweat pores are closely related to the dispersion of lungs. Therefore, treatment of skin lesions can start from opening the inhibited lung Qi. Acupuncture at the LU10 could stimulate blood and energy circulation in lung meridian, resulting in wind disappearance and expected curative effects.^[13]^

It is believed in modern medicine that the causes of urticaria are complicated, which are normally exogenous and endogenous according to different sources.^[14]^ At present, mainstream scholars are convinced that the activated mast cells induced by the immunologic and nonimmunologic mechanisms are the key effector cells in urticaria pathogenesis. Abnormal activation of the coagulation system is also believed to be involved in urticaria onset.^[15–17]^ It has been abundantly reported in recent literatures that acupuncture and moxibustion are effective in treating urticaria.^[18,19]^ Acupuncture can activate endomorphine-like substances, inhibit and block the transmission of pain and itching signals, thus realizing the analgesia and antipruritic effects,^[20]^ regulating the immune system and inhibiting allergic reactions.^[21]^ Scholars have found that acupuncture treatment of urticaria shows bidirectional regulation on the immune system, enabling it to work in the normal or optimal status.^[22]^

Restricted by a single case without a long-term follow-up, large-scale randomized clinical trials shall be conducted in the future. As the 1st report on acute urticaria treatment via acupuncture at the Yuji (LU10), this research could provide guidance for our future research, which will be centered on the molecular biologic mechanism of acupuncture treatment of acute urticaria.

## Author contributions

**Conceptualization:** Mingxin Xue.

**Writing – original draft:** Shuai Zhou, Ruirui Zhao.

**Writing – review & editing:** Shuai Zhou, Ruirui Zhao.
